# Echocardiographic Assessment of the Pulmonary Vein to Pulmonary Artery Ratio in Canine Heartworm Disease

**DOI:** 10.3390/ani13040703

**Published:** 2023-02-17

**Authors:** Jorge Isidoro Matos, Alicia Caro-Vadillo, Yaiza Falcón-Cordón, Sara Nieves García-Rodríguez, Noelia Costa-Rodríguez, Elena Carretón, José Alberto Montoya-Alonso

**Affiliations:** 1 Internal Medicine, Veterinary Medicine and Therapeutic Research Group, Faculty of Veterinary Medicine, Research Institute of Biomedical and Health Sciences (IUIBS), Universidad de Las Palmas de Gran Canaria (ULPGC), 35016 Las Palmas de Gran Canaria, Spain; 2 Hospital Clínico Veterinario, Faculty of Veterinary Medicine, Universidad Complutense de Madrid (UCM), 28040 Madrid, Spain

**Keywords:** *Dirofilaria immitis*, heartworm disease, pulmonary vein, pulmonary artery, PV:PA ratio, RPAD index, pulmonary hypertension, animal diseases

## Abstract

**Simple Summary:**

Pulmonary hypertension (PH) is a phenomenon frequently seen in dogs with heartworms. Given the seriousness of this condition, numerous studies have focused on determining its presence. In this study, the pulmonary vein to pulmonary artery ratio (PV:PA ratio), determined by echocardiography, is evaluated in 151 dogs to determine its usefulness in the detection of PH in heartworm. The results showed that the PV:PA ratio could be useful as a complementary diagnostic method to estimate the presence of moderate or severe PH in these patients.

**Abstract:**

**Background:***Dirofilaria immitis* produces proliferative pulmonary endarteritis and pulmonary thromboembolism in infected dogs. The pulmonary vascular lesions lead to irreversible and persistent structural damage and, as a consequence, sustained precapillary pulmonary hypertension (PH). The purpose of this study was to assess the diagnostic value of the pulmonary vein to pulmonary artery ratio (PV:PA ratio) to determine moderate or severe PH (>50 mmHg) in dogs with heartworm disease. Methods: A total of 151 naturally heartworm-infected and 66 healthy dogs were included in the study. The presence/absence of PH was based on the right pulmonary artery distensibility index (RPAD index < 29.5%), and the PV:PA ratio was echocardiographically measured by the time–motion mode (M mode) and two-dimensional mode (2D mode). Other echocardiographic parameters were also assessed (pulmonary trunk to aorta ratio, tricuspid regurgitation pressure gradient, and AT:ET ratio). Results: The results of the PV:PA ratio showed a highly positive correlation between the M and 2D modes (r = 0.928). The PV:PA ratio obtained by the M mode was identified as the strongest predictor for RPAD index (R^2^ 0.628, *p* < 0.0001) with a good diagnostic accuracy (AUC = 0.99). The results of PV/PA by the 2D mode showed a similar prediction for the RPAD index (R^2^ 0.606, *p* < 0.0001) with a good diagnostic accuracy (AUC = 0.98). Both of the 2D and M modes’ PV:PA ratios decreased significantly with the presence of PH. A cut-off value of ≤0.845 showed high sensitivity and specificity for the M mode (97% and 94%, respectively) and the 2D mode (96% and 93%, respectively). Conclusions: The PV:PA ratio may be useful as a complementary diagnostic method for the estimation of moderate or severe PH in dogs with heartworm.

## 1. Introduction

Canine heartworm is a serious disease caused by *Dirofilaria immitis*, which is transmitted through the bite of vector mosquitoes and mainly affects canids and felines, both domestic and wild. Direct contact of *D. immitis* adults with the intimal layer of pulmonary arterial vessels causes irreversible proliferative pulmonary endarteritis, with a reduction in the vascular lumen and a loss of elasticity of the pulmonary arteries [1]. In addition, there is an obstruction to blood flow due to the presence of parasites in the pulmonary arteries and the consequent formation of thrombi and emboli [2]. These events chronically lead to increased resistance to blood flow, which in turn increases pressure in the pulmonary arteries, as is defined as precapillary pulmonary hypertension (PH) [3]. PH in dogs with heartworm is a common, serious, and life-threatening condition, and many of the clinical signs seen in infected dogs are due to the presence of PH [4].

Currently, echocardiography provides the most accurate, non-invasive, available, and cost-effective estimate of pulmonary arterial pressure in dogs. The right pulmonary artery distensibility Index (RPAD index) was previously validated as a valuable and useful method to estimate the presence of PH in dogs infected with heartworm disease [2,5] and has also been shown as an effective method for the diagnosis of PH due to other causes, especially when it is not possible to assess tricuspid or pulmonary regurgitation flows [6]. However, new effective and useful echocardiographic parameters should be implemented to estimate the severity of PH due to the high presentation of this phenomenon in dogs infected by *D. immitis* [4].

The structural changes observed in the pulmonary vasculature are a reflection of cardiorespiratory hemodynamic alterations. Evaluation of pulmonary vein (PV) diameter and pulmonary artery (PA) diameter in dogs and cats has been shown to be a valuable echocardiographic index. The PV:PA ratio has been useful in the diagnosis of pulmonary venous congestion in the feline and canine species derived from left heart failure [7]. In addition, the application of the PV:PA ratio in the identification of PH has been validated in dogs suffering from idiopathic pulmonary fibrosis in the West Highland terrier breed [8], and in other pathologies that produce precapillary PH due to pulmonary or thromboembolic pathologies [9]. Therefore, the aim of this study was to determine if PH in dogs with heartworm can be assessed using the PV:PA ratio, and to evaluate its accuracy compared to other indices to estimate PH.

## 2. Methods

### 2.1. Study Animals

In the present prospective study, 217 client-owned dogs presented to the Veterinary Teaching Hospital of the University of Las Palmas de Gran Canaria (Canary Islands, Spain), between September 2020 and July 2022, were included. The dogs lived in a hyperendemic area of *D. immitis* [10]. A complete record was kept for each animal, including identification (age, sex, breed, and weight), clinical history, and demographic data. Of them, 69.6% (151/217) were diagnosed with heartworm infection using a commercial immunochromatographic test kit (Urano test Dirofilaria^®^, Urano Vet SL, Barcelona, Spain), while 30.4% (66/217) were considered healthy based on the absence of clinical signs according to history, physical examination, cardiovascular assessment, echocardiographic evaluation, and the negative results of the *D. immitis* antigen detection test. Dogs that had previously received any cardiovascular medication were not considered candidates for the study. Likewise, animals that showed clinical signs and echocardiographic evidence of cardiac conditions other than heartworm disease (i.e., left heart disease, dilated cardiomyopathy, congenital diseases) were excluded from the study. All owners were informed and gave consent to participate in the study.

Infected dogs were considered as symptomatic when presence of one or more symptoms related to heartworm disease (dyspnea, cough, exercise intolerance, weakness, loss of weight and syncope) was observed. In addition, the occurrence of ascites, jugular venous distension, and hepatomegaly were also observed in dogs with right-sided congestive heart failure (R-CHF). Echocardiographic examinations were performed on all animals prior to the beginning of adulticide treatment.

### 2.2. Echocardiography

Dogs were subjected to an echocardiographic examination, using an ultrasound equipment with spectral and color Doppler and multifrequency probes (2.5–10 MHz, Viviq Iq^®^, General Electric, Boston, MA, USA). All dogs were conscious, sedation-free, and under electrocardiographic monitoring during the whole test. For each measurement, three continuous cardiac cycles were recorded. All echocardiographic recordings were made by the same cardiologist.

The absence or presence of PH, as well as the severity, was based on the determination of the RPAD index as previously described [2,6], and an index < 29.5% correlated with moderate or severe PH (>50 mmHg) [6,11]. According to clinical status and the results of the RPAD index, dogs were divided into three groups: Group A included healthy dogs, all of them with absence of PH. Group B consisted of heartworm-infected dogs and absence of PH. Group C comprised heartworm-infected dogs with PH.

**Figure 1:**
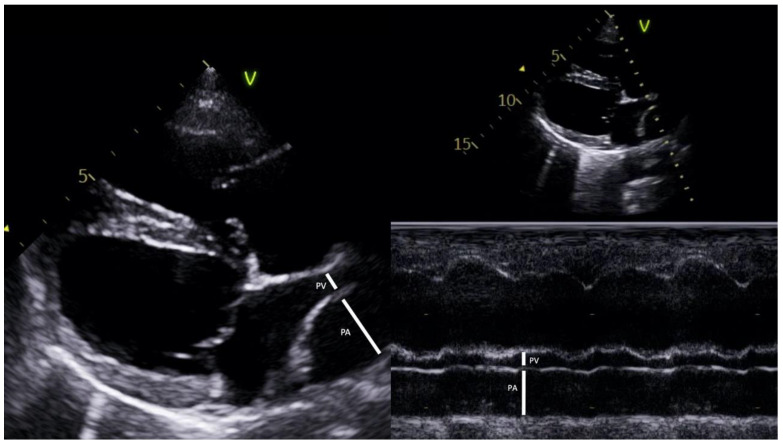
The image shows representative measurement and calculation of the pulmonary vein (PV) to pulmonary artery (PA) ratio in two-dimensional (2D) and time–motion mode (M mode) echocardiography. PV:PA ratio (2D and M mode) was obtained in a heartworm-infected dog with an RPAD index < 29.5% (Right parasternal long axis four-chamber view).

For measurement of the PV:AP ratio, a 4-chamber view of the right parasternal long axis was optimized to simultaneously visualize a longitudinal section of the right ostium of the right cranial pulmonary veins and the transverse section of the right pulmonary artery branch. Measurements of PV and PA diameters were taken in M (time–motion) and in 2D (two-dimensional) modes as previously described and validated [8,9]. For both measurements, the inner-edge-to-inner-edge method was used with timed measurements at the end of the T wave (end-systole), drawing a line perpendicular to the medial PV and passing through the center of the adjacent right PA at the site of maximum vascular diameter (Figure 1) and 3 measurements were averaged. The interobserver variability of PV:PA image acquisition and PV:PA measurement was evaluated separately in 10 dogs. In order to demonstrate the presence/absence of PH as well as severity in the animals analyzed, the following echocardiographic parameters were also determined: pulmonary trunk to aorta ratio (PT:Ao ratio), tricuspid regurgitation pressure gradient (TRPG), and the velocity of the outflow tract of the right ventricle through the relationship between acceleration times (AT) and ejection times (ET) using the AT:ET ratio.

On the other hand, the presence of visible parasites in the pulmonary arteries and right heart chambers was assessed according to Venco et al., 2003 [12]. From low to high burden: parasite burden 1 was determined when the parasites were not observed, parasite burden 2 when they were observed in the right pulmonary branch, parasite burden 3 when they were observed in the pulmonary trunk, and parasite burden 4 when adult parasites were observed in the right heart chambers (caval syndrome).

### 2.3. Statistical Analysis

Statistical analyses were performed using commercially available software (BM SPSS^®^ Statistics 25.0, New York, NY, USA). For categorical variables, frequencies and percentages are shown. The differences in parameters between groups were evaluated with Pearson’s non-parametric Chi^2^ test and, just in the case of 2 × 2 tables, Fisher’s exact test was applied. For continuous variables, the differences in the parameters between groups were evaluated by means of Mann–Whitney/Kruskal–Wallis tests (non-parametric) or T-student/ANOVA (parametric) based on the normality of the variables to be evaluated by means of Shapiro–Wilks test. When significant differences were identified, post hoc pairwise comparisons were made using Pearson’s *p* test with Bonferroni corrections. The results of the statistical procedures with respect to PV:PA ratio were also graphed by scatter plot. A simple linear regression was performed between the RPAD index values and the PV:PA ratio. To identify the best one-variable model, a regression analysis of all subsets was performed with a maximum improvement in R^2^ as a selection criterion. Receiver operator characteristic curve (ROC) analyses were performed to determine the optimal cut-off values for the prediction of RPAD index < 29.5% (moderate or severe PH). For all analyses, *p* < 0.01 was considered statistically significant.

## 3. Results

Detailed epidemiological and clinical results of the studied dogs are summarized in Table 1. According to the results obtained, group A included 66/217 (30.4%) dogs, group B consisted of 81/217 (37.3%) dogs, and group C comprised 70/217 (32.3%) dogs. In this study, 28 different breeds were evaluated, with mongrel dogs (46.1%), Canary hound (8.3%), and American pit bull terrier (5.1%) being the main breeds analyzed. No statistical differences in age, body weight, and number of males/females were found between the three groups. Based on the RPAD index, PH was present in 46.36% of dogs infected by *Dirofilaria immitis*. The presence of symptoms was significantly higher in dogs from group C (87.1%) than in groups B (30.9%) and A (0%) (*p* < 0.01). Dogs with R-CHF were only reported in group C (*p* < 0.01). The most common clinical sign found was cough (50%), followed by exercise intolerance (21.0%), cyanosis (10.3%), dyspnea (8.5%), syncope (6.2%), and weight loss (1.3%).

**Figure 2:**
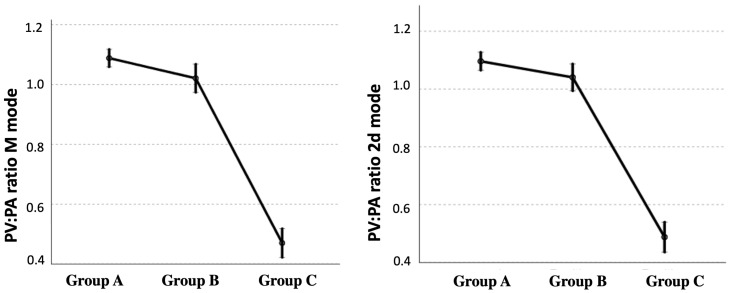
Scatterplots illustrating the right pulmonary vein to pulmonary artery (PV:PA) ratios in time–motion mode (M mode) and two-dimensional mode (2D mode), obtained in healthy dogs (group A), in dogs with heartworm and absence of PH (group B), and in dogs with heartworm and presence of PH (group C). The results show the estimated marginal means and their CI 95%.

The results of the echocardiographic parameters measured in each group are described in Table 1. The PV:PA ratio, measured by both the 2D mode and the M mode, decreased significantly with the presence of PH (Figure 2). Echocardiographic data showed that this decrease was mainly due to an increase in the size of the PA, compared with the diameter of the PV. A decrease in the AT:ET ratio was also observed, as well as an increase in the variables PT:Ao ratio and TRPG, related to the presence of PH (Table 1). When the parasite burden was assessed, healthy dogs obviously showed an absence of adult parasites, while parasite burden was higher in dogs from group C than in dogs from group B. All dogs from group C showed the echocardiographic presence of heartworms (scores 2 to 4), while no worms were echocardiographically visible in 8.6% (7/81) of dogs from group B (score 1).

**Figure 3:**
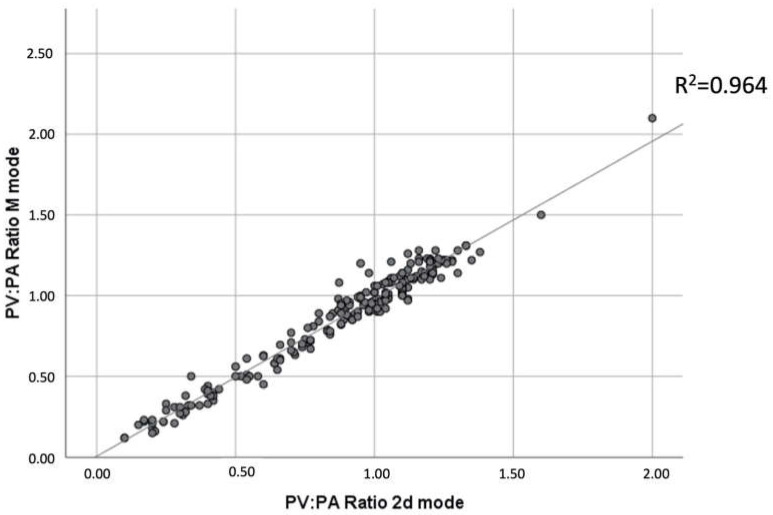
Linear regression illustrating right pulmonary vein to pulmonary artery (PV:PA) ratios in motion mode over time (M mode) and two-dimensional mode (2D mode). The M and 2D modes of the PV:PA ratios were highly positively correlated (r = 0.928). The correlation was significant at the 0.01 level (bilateral).

Furthermore, the coefficient of determination (R^2^) of the regressions that allow comparing the quality of the PV:PA relationship, in the M and 2D modes, with respect to the RPAD index, were studied. The results showed that the M and 2D PV:PA ratios were highly positively correlated (r = 0.928) (Figure 3). Furthermore, these two ratios were positively correlated with the RPAD index with r between 0.788 and 0.774, and R^2^ of 0.628 and 0.606, respectively (Table 2).

The results of the ROC curves of the PV:PA ratio measurement in the M mode and in the 2D mode to estimate the event of suffering from PH (RPAD index < 29.5%), and the cut-off points of the parameters that maximize sensitivity and specificity (through the Youden index) are shown in Table 3. The AUCs of the two ratios were excellent (>0.9). For the PV:PA ratio in the M and 2D modes, any value ≤ 0.845 was suggestive of the presence of PH in 97% and 96%, respectively, and a value > 0.845 was suggestive of the absence of PH in 94% and 93%, respectively.

## 4. Discussion

PH is a serious, frequent, and non-reversible condition in dogs with heartworm disease [5,13]. The clinical signs that these dogs present are often due to the progression of PH, which, in the final stages, can lead to the development of R-CHF. Therefore, determining the presence of PH using effective methods is essential in heartworm disease since they will provide information about the chronicity of the disease, and will help establish the most accurate treatment protocol and prognosis.

The parasite load is also normally evaluated in the study of PH in dogs parasitized by *D. immitis* [2,4,6]. Although, in this study, burden was higher in dogs with PH, the influence of the parasite load in the development of proliferative pulmonary endarteritis and subsequent PH was not clear, and several authors have stated that the chronicity, immune reaction, and the lifestyle of the infected dog (animals that exercise frequently), have a more notable influence on the development of damage at the vascular level. However, a high parasite load has been shown to be subjective in increasing the proportion of moderate or severe HP in the infected dogs [4,6].

Currently, the RPAD index is used as the gold standard for determining the presence and severity of PH in dogs infected by *D. immitis*, and its utility in accurately estimating PH in these dogs has been demonstrated [1,2,5,6,13]. However, new diagnostic methods should continue to be studied to help in the measurement of PH in dogs with heartworm. In this study, the analysis of the other indirect echocardiographic predictors of PH in canine heartworm reported a significant increase in the PT:Ao ratio and the TRPG, as well as a decrease in the AT:ET ratio in proportion to the presence of PH, which supports the previously published studies [14,15,16]. Moreover, the results of this study demonstrated the validity of the PV:PA ratio to determine the presence of PH in dogs with heartworm disease, as the results in the PV:PA ratio showed significant variations depending on the presence/absence of PH. The lower values of the PV:PA ratio found in dogs with heartworm with PH may be as a result of the pulmonary artery distention caused by the chronic proliferative endarteritis resulting from the direct contact of the parasites with the intima layer of the pulmonary artery [17,18]. Likewise, significantly lower PV:PA ratio values have been found in dogs with precapillary PH caused by other pathologies, in comparison to animals without PH [9]. These findings suggest that the PV:PA ratio is an adequate tool to determine the presence of PH, regardless of the pathology that produced the increase in pulmonary arterial pressure.

In this study, both measurements of PV:PA ratio—the M and 2D modes—highly correlated, corroborating that both can be accurately used to study the presence/absence of PH in dogs infected by *D. immitis*. These results are similar to those reported in a previous study, using both the M and the 2D modes, which also found no significant variations between the two methods in healthy dogs [19]. Although, in the present study, the PV:PA ratio in the M mode showed slightly more accurate values in the estimation of an RPAD index < 29.5% in dogs with heartworm, the results showed that both measurements can be safely used.

In this study, an average value of PV:PA ratio of approximately 1.0 was found in healthy dogs, similar to values previously described by other authors [19,20]. Furthermore, a cut-off point of 0.845 was described for both the M and 2D modes to estimate pulmonary pressures > 50 mmHg, as correlated with an RPAD index < 29.5%. Recently, other authors studied the PV:PA ratio for the estimation of precapillary PH caused by pathologies other than heartworm disease, using the M and 2D modes, and reported a cut-off point of <0.70 (sensitivity 96%, specificity 82%) for the estimation of moderate PH (TRPG > 50 mmHg) [8,9]. The PV:PA ratio has also been used to estimate the survival of West Highland terrier dogs that presented pulmonary fibrosis and developed PH using the cut-off level of <0.70, demonstrating the usefulness of the measurement performed in the 2D mode and the M mode [9]. The cut-off value of <0.70 reported by these authors is lower than that observed in this study, which may be due to the difference in the sample size (67 vs. 217 dogs), the echocardiographic method established to estimate the presence of PH (TRPG vs. RPAD index), or the different pathologies studied by other authors (angiostrongylosis, bronchomalacia, precapillary PH of unknown origin, brachycephalic syndrome, pulmonary thromboembolism, eosinophilic bronchopneumopathy, etc.). Another considerable difference is the average weight of the animals studied, which is considerably higher in the animals suffering from heartworm disease compared to the study by Roels et al. (2019), (7.4 kg vs. 18.65 kg) [9], and the precise echocardiographic measurement of the vascular structures is more difficult to determine in small dogs compared to heavier animals [21]. Finally, sensitivity and specificity varied between studies, both being higher in the present study.

The use of the PV:PA ratio is an easy, fast, and precise way to determine the hemodynamic status in dogs with heartworm, being a good option for inexperienced sonographers. Since the variation in the diameter of these vessels is a direct reflection of the pathology, the determination of the PV:PA ratio allows the severity of the disease to be established. Moreover, it is a very reliable intraoperative method and is a useful tool to assess patient follow-up. In the same echocardiographic view (right parasternal long axis four-chamber view) it is possible to visualize the presence of parasites and measure the RPAD index, so both measures complement each other, and it is possible to compare results in the same image and cardiac cycle.

The main limitation of this study is that the animals did not undergo right heart catheterization for the determination of pulmonary arterial pressure, which is considered the gold standard method, but instead underwent transthoracic echocardiography, considered an alternative tool to estimate the presence of PH. In this sense, the only echocardiographic measure used in this study to estimate the presence of PH in dogs with heartworm disease was the RPAD index, which, despite having shown an excellent correlation with the invasive pulmonary artery pressure measurement [6], presents limitations [3]. Therefore, to confirm the usefulness of the PV:PA ratio to determine the presence of PH, it is necessary to carry out new studies using other parameters for estimating the presence of PH, in addition to a larger sample size, which would allow standardizing protocols and obtaining objective reference values to satisfactorily establish the diagnosis and severity of PH in dogs with heartworm.

## 5. Conclusions

The echocardiographic measurement of the PV:AP ratio was able to discriminate between dogs without PH—being healthy or infected by *D. immitis*—and heartworm-infected dogs with PH (RPAD index < 29.5%). Therefore, the PV:PA ratio may be a useful tool to echocardiographically evaluate vascular status in patients with heartworm disease. The PV:PA ratio ≤ 0.845, both in the M and 2D modes, suggested an acceptable cut-off value to differentiate between dogs with PH and dogs without PH.

## Figures and Tables

**Table 1:** Clinical, epidemiological, and echocardiographic parameters of the studied dogs (group A: healthy dogs, group B: heartworm-infected dogs and absence of PH, group C: heartworm-infected dogs with PH). Data represent median and standard deviation (min–max) unless otherwise stated: RPAD index, right pulmonary artery distensibility index; TRPG, peak tricuspid regurgitation systolic pressure gradient; PT, pulmonary trunk; Ao, aorta; PV:PA pulmonary vein to pulmonary artery ratio; M mode, time–motion mode; 2D mode, two-dimensional mode; AT:ET, right acceleration time to ejection time ratio. Results for female, respiratory symptoms, and right-sided CHF are expressed as *n* (%). Results for parasite burden are expressed as median (range). Significant differences were found when *p*-value < 0.01. (*): significant differences observed between groups C and B; (ⱡ): significant differences observed between groups C and A; (●): significant differences observed between groups B and A.

Clinical, Epidemiological, and Echocardiographic Parameters.	All Dogs (*n* = 217)	Group A (*n* = 66)	Group B (*n* = 81)	Group C (*n* = 70)	*p*-Value
Body weight (kg)	18.04 ± 11.24	16.76 ± 11.15	17.90 ± 12.35	18.65 ± 12.83	0.23
Age (years)	7.17 ± 4.66	8.08 ± 5.83	6.53 ± 4.08	8.21 ± 5.51	0.47
Female: number (%)	116 (53.5%)	30 (45.5%)	50 (61.7%)	36 (50.4%)	0.46
Respiratory symptom (%)	86 (39.6%)	0 (0.0%)	25 (30.9%)	61 (87.1%)	0.00 ^(*,ⱡ)^
Right–sided CHF (%)	26 (12.0%)	0 (0%)	0 (0%)	26 (37.1%)	0.00 ^(*,ⱡ)^
TRPG (mmHg)	21.05 ± 33.12	4.42 ± 3.11	3.95 ± 2.43	56.51 ± 39.18	0.00 ^(*,ⱡ)^
RPAD index (%)	34.80 ± 11.52	42.11 ± 5.04	40.08 ± 6.88	20.62 ± 6.82	0.00 ^(*,ⱡ)^
PT:Ao ratio	1.05 ± 0.18	0.96 ± 0.07	0.95 ± 0.15	1.26 ± 0.15	0.00 ^(*,ⱡ)^
PV:PA ratio (M mode)	0.86 ± 0.33	1.08 ± 0.12	1.03 ± 0.20	0.47 ± 0.21	0.00 ^(*,ⱡ)^
PV:PA ratio (2D mode)	0.88 ± 0.33	1.09 ± 0.13	1.05 ± 0.20	0.48 ± 0.23	0.00 ^(*,ⱡ)^
AT:ET	0.33 ± 0.09	0.37 ± 0.06	0.38 ± 0.07	0.24 ± 0.06	0.00 ^(*,ⱡ)^
Parasitic burden (1–4)	2.00 (1–4)	0.00 (0–0)	2.00 (1–3)	3.00 (2–4)	0.00 ^(ⱡ,●)^

**Table 2:** Results of simple regression analyses for the prediction of RPAD index < 29.5%. CI 95%: confidence interval 95%; *p*: Pearson correlations; PV:PA: pulmonary vein to pulmonary artery ratio; R^2^: coefficient of determination; RPAD index: right pulmonary artery distensibility index.

Method	R^2^	*p*-Value	CI 95%	Regression Equation
PV:PA ratio (M mode)	0.628	<0.0001	(24.654, 30.328)	RPAD index = 10.634 + 27.492 × PV:PA ratio (M mode)
PV:PA ratio (2D mode)	0.605	<0.0001	(23.914, 29.711)	RPAD index = 10.809 + 26.812 × PV:PA ratio (2D mode)

**Table 3:** Sensitivity, specificity, and Youden index of cut-off points of PV:PA for predicting PH estimation of right pulmonary artery distensibility index (RPAD index) < 29.5%. AUC: area under receiver operating characteristic curve; CI 95%: confidence interval 95%; *p*: Pearson correlations; PV:PA: pulmonary vein to pulmonary artery ratio; Se: sensitivity; Sp: specificity.

Method	AUC	CI 95%	*p*-Value	Cut-off	Se	Sp	Youden Index
PV:PA ratio (M mode)	0.988	(0.967, 1.000)	<0.0001	≤0.845	0.97	0.94	0.91
PV:PA ratio (2D mode)	0.983	(0.970, 1.000)	<0.0001	≤0.845	0.96	0.93	0.89

## Data Availability

The raw data supporting the conclusions of this article will be made available by the authors, without undue reservation.
